# Supplementary material for: Are carbon emissions trading and green financial instruments synergistic? -Comprehensive quantitative research based on content analysis
Source: PLoS One. 2024 Mar 7;19(3):e0298601. doi: 10.1371/journal.pone.0298601 (PMC10919601; doi:10.1371/journal.pone.0298601)
Supplement: S1 Data — (ZIP) [file pone.0298601.s001.zip › Data/地级市Z_F工作报告.zip!/╦─┤¿/─╧│Σ╩╨/└┤╫╘╣1⁄2╓┌║┼í╛┬φ┐╦╩2╛▌═°í┐_2019.docx]

各位代表：

　　现在，我代表市人民政府，向大会报告工作，请予审议，并请市政协委员提出意见。

一、2018年工作回顾

　　过去一年，是南充发展开拓奋进、强势突破的一年。一年来，我们坚定以习近平新时代中国特色社会主义思想为指导，深入学习贯彻党的十九大精神和习近平总书记对四川工作系列重要指示精神，在市委的坚强领导下，在市人大、市政协的监督支持下，牢牢把握稳中求进工作总基调，认真落实高质量发展要求，紧紧围绕建设成渝第二城、争创全省经济副中心战略目标，大力实施“155发展战略”，打好打赢“九场战役”，全面完成各项目标任务，经济社会发展取得新成绩。全市实现地区生产总值2006亿元、在川东北率先迈入“2000亿俱乐部”，增长9%、创5年来新高，增速居全省第3位；全社会固定资产投资1674.7亿元、增长15.5%，增速居全省第3位；一般公共预算收入113.9亿元，增长10.3%；规上工业增加值增长11%、居全省第3位；社会消费品零售总额960亿元、增长12.4%，增速居全省第2位；城镇居民人均可支配收入30810元、增长8.7%，增速居全省第3位；农村居民人均可支配收入13583元、增长9.6%，增速居全省第5位；主要经济指标增速继续保持全省“一方阵”，争先进位势头更加强劲。

　　一年来，我们主要完成了以下工作：

　　（一）着力扩规模增效益，产业集群加速壮大。聚焦“5+5”重点产业，精准发力、综合施策，推动产业规模和质效不断提升。汽车汽配产业加快崛起。实现产值701亿元、增长16.2%。吉利一期产能不断释放、销售整车5500余台，中国重汽、顺发机械等项目竣工投产，吉利二期、重庆银翔、博萨汽配等项目加快推进。油气化工产业提速发展。实现产值352亿元、增长25.1%。四川能投PTA项目投产在即，联成化学、石达化工等项目达产达效，青龙化工、鑫安邦定等项目加快建设。丝纺服装产业持续振兴。实现产值450亿元、增长13.1%。家丰时装、银海丝绸蚕桑基地等项目竣工投产，富安娜家纺、依格尔纺织产销两旺。南充“中国绸都”称号通过复审。现代物流产业质效双升。实现产值455.8亿元、增长16.6%。中国物流南充分中心、南充公用型保税仓、南鑫钢材城等项目建成运营，南充粮食物流城、花都商贸物流中心等项目加快建设，浙江传化、友信崧锋等企业不断壮大。现代农业产业率先突破。产值跨过千亿大关、达到1020亿元、增长10.2%。坚持区域化、流域化、全域化布局，集中连片发展特色优势产业，新增晚熟柑桔基地25万亩、木本油料基地2.3万亩，新建成万亩级现代循环农业园区20个。全年认证“三品一标”农产品41个、有机农产品基地5.2万亩。阆中五龙人文田园度假区、南部八尔湖乡村旅游区、营山“耕读原乡”产业园等一批农旅结合项目惊艳亮相。升钟灌区二期、金鸡沟水库等水利工程加快实施，新增高标准农田36.7万亩。“五大百亿战略性新兴产业”蓬勃兴起。实现产值258亿元、增长13.8%。电子信息产业快速集聚，三环七期、恒诺电子等项目建成投产，中科九微、和泰光纤一期等项目开工建设，暴风启智成功创建省级新型信息消费示范项目。新材料产业扎实推进，鑫达一期达产达效，中远一期、旭阳碳纤维等项目竣工投产，家旺钢化、中城建新型建筑材料等项目稳步实施。高端装备制造产业特色鲜明，九天真空军工配套、镱发不锈钢等项目建成投产，思杰聚典智能、精丰机械等项目加快实施。生物医药产业不断壮大，康美滕王阁药业、九威阁中药材等项目竣工投产，香雪制药二期等项目有力推进。节能环保产业加快发展，污水处理与废水资源化、固体废弃物处理等一批节能环保技术得到推广应用，嘉源环保、国能生物、鑫宇源环保等项目有序实施。

　　（二）着力稳投资强支撑，项目攻坚全面发力。紧扣“5+100”重大工程项目，扭住不放、抓紧抓牢，全年完成项目投资1338.7亿元、增长10.5%；南充被评为全省年度投资和重点项目推进工作成绩突出单位，获得省政府通报表扬。城市建设项目强力推进。清泉坝双层互通、西华路下穿等项目竣工通车，果城路、育英路、白土坝路、西河北路等城市道路综合改造全面完成，金融广场、火车北站站前广场等城市地标全新亮相，“印象嘉陵江”湿地公园、缤纷水岸等城市会客厅靓丽呈现，南充城市夜景亮化工程刷爆“朋友圈”、春节期间连续登上央视新闻。交通枢纽项目有序实施。成南达万高铁、汉巴南铁路、南充动车所前期工作取得重要进展，南充成为西部首个开行始发至北京、上海高速动车组的地级市。绵西高速、定升公路建成通车，都京港嘉陵江大桥主体完工，营达高速公路、顺蓬营一级公路加快建设。高坪机场年旅客吞吐量达到94万人次。嘉陵江航运配套二期基本建成。教科文卫项目齐头并进。新（改、扩）建中小学（幼儿园）校舍、运动场86万平方米，南充科技职业学院一期全面完工，嘉陵江文化旅游职业学院加快建设，四川电影技术职业学院成功签约。吉利新能源汽车研究院完成组建。嘉陵江文化综合保护开发加快实施，营山文化中心、蓬安文体中心等项目扎实推进。市妇幼保健院新院区、市中心血站检验大楼建成投用。旅游三产项目加快建设。凤垭山天乐谷正式开园，阆中水城、朱德故里客家民宿博览园、蓬安相如故城保护开发等文化旅游项目加快推进；全市新增3A级景区6个，文化旅游产业产值达到750亿元、增长17%。仁和春天、尚品时代广场等项目投入运营，川东北金融中心一期基本建成；全市实现服务业增加值800.1亿元、增长10.5%，增速居全省第1位。产业园区项目统筹推进。南充新能源汽车产业园初具规模，南充经开区加快转型升级，全市新增省级经开区5个，工业园区建成面积达到110平方公里、工业集中度提升至80%。国家农业科技园区通过科技部验收，中法农业科技园试开放，国家现代农业示范区提质增效，西充农村产业融合发展示范园成功跻身首批国家示范园。南充现代物流园商家入驻率提高到92%。“100件大事”全面铺开。坚持把“100件大事”作为牵引带动全局工作的“牛鼻子”，按照工程化、项目化、具体化的要求，细化分解支撑项目229个，逐一挂出作战图、下达任务书、列出时间表，推动完成年度投资1100亿元。其中，市本级22个支撑项目完成投资45.2亿元、占年度计划的102.5%，9县（市、区）207个支撑项目完成投资1054.8亿元、占年度计划的104.5%；黄金江岸一期、营山中医医院等17个支撑项目全面完工。

　　（三）着力抓改革促开放，动力活力持续激发。以庆祝改革开放40周年为契机，不断深化改革、扩大开放，推动经济内生动能加速释放。重点改革有序推进。供给侧结构性改革深入实施，淘汰落后产能企业101户，非住宅商品房去库存周期缩短了13个月，企业和居民用能成本减少2.16亿元。农业农村、行政审批等改革取得实质性进展，公立医院薪酬制度改革等6项国、省改革试点全面完成，商事制度改革不断深化。创新驱动支撑有力。建立首批市级院士（专家）工作站2个。顺庆工业集中区成功创建为省级高新区。创业小镇二期扎实推进，南充高新孵化园二期基本建成，西华师范大学科技园孵化能力持续增强，全市孵化器面积达到71.7万平方米。595户科技型中小企业通过国家认证，总量居全省第3位。全年专利申请量3220件、增长15%，专利授权量1442件、增长46%，研发投入占GDP比重提高0.12个百分点，科技对经济增长贡献率超过50%。成功创建全国消费品工业“三品”战略示范城市。开放合作全面拓展。务实推动成南协作、融渝发展和川东北经济区市际合作，成功举办第九届C21城市论坛和深圳、杭州、香港等地专题招商活动，全年新签约项目269个、增长29.3%，实现到位资金680.7亿元、增长17.2%，5个“百亿级”项目签约落地。与美国托莱多市缔结协议，正式建立国际友好城市关系。民营经济加快发展。精心筹备召开高规格的民营经济健康发展暨首届南商发展大会，出台“1+4”政策包，鼓励支持引导民营经济健康发展，全市新增各类市场主体4.2万户，实现民营经济增加值1220.7亿元、占地区生产总值比重达60.9%。南充获评全省促进民营经济发展先进市。

　　（四）着力强功能提品质，人居环境明显改善。 统筹推进城乡区域协调发展，城乡面貌实现新提升。新型城镇化步伐加快。全市常住人口城镇化率提高1.67个百分点、达到48.14%。中心城区建成区面积145平方公里、常住人口137万人；燕儿窝、五里店等旧城改造加快推进，下中坝、清泉坝、白马湖等城市新区功能配套更加完善，双女石公园、丝绸文化主题公园等一批城市公园建成开园。阆中入选国际可持续发展试点城市，南部被评为“四好农村路”全国示范县；23个“百镇建设行动”试点镇建设取得新进展。乡村振兴开局破题。建成幸福美丽新村560个。新（改）建农村公路2678公里，完成渡改桥17座，新增村级综合文化服务中心423个。农村集体产权制度改革全面启动，全市所有行政村完成清产核资。培育新型农业经营主体898个，总量达到1.2万个。环境质量稳步提升。主城区PM2.5年均浓度较基准年下降21.3%，空气质量优良天数率达到80.4%、较基准年提高6个百分点；河（湖）长制全面落实，嘉陵江干流、全市重点湖泊保持在Ⅱ类水质；农用地土壤污染状况详查全面完成。中心城区、县城污水集中处理率分别提高到90.2%、83.8%，生活垃圾无害化处理率均达100%。中、省环保督察及“回头看”反馈问题整改有力有序推进。全市森林覆盖率达到40.64%。单位GDP能耗下降5.94%。

　　（五）着力办实事惠民生，社会大局保持稳定。认真践行以人民为中心的发展思想，持续加大民生投入，群众生活水平不断提高。脱贫摘帽决战全胜。仪陇、高坪、嘉陵成功摘帽，阆中、营山顺利完成省级评估验收，全市423个贫困村成功退出、7.8万贫困人口实现脱贫。新建成脱贫奔康产业园538个，带动3.5万贫困户长效增收。完成易地扶贫搬迁3.25万人、农村危房改造4万户、地灾避险安置690户，解决9万建档立卡贫困人口饮水安全问题。资助贫困家庭学生26.4万人次，贫困人口县域内住院和慢性病门诊医疗费用个人支付占比控制在10%以内。深化与金华、温州、台州3市扶贫协作，签订合作协议112个，实施帮扶项目32个。民生保障更加有力。十项民生工程、20件民生实事全面完成。营山嘉陵江引水工程等重大民生项目加快实施，全市新（改）建公厕318座，完成城镇危旧房棚户区改造4.5万套、土坯房整治改造7.3万户。社会保障体系不断健全，城乡低保标准稳步提高，“五大保险”参保1125.5万人次，新增城镇就业8万人，城镇登记失业率控制在3.8%以内。退役军人服务管理和农民工服务保障得到加强。社会救助体系更加健全。社会事业全面进步。西充、营山通过全国义务教育发展基本均衡县评估认定，全市学前三年入园率达89%，高中阶段毛入学率达95%，职业教育、民办教育、特殊教育、继续教育加快发展。全市新增医疗床位1500张，在全省率先实现城镇职工医保异地结算。成功举办第六届嘉陵江合唱艺术节、第二届落下闳春节文化博览会等重大活动，川北大木偶亮相平昌冬奥会闭幕式，南充文化影响力进一步提升。

　　（六）着力筑防线守底线，风险防控扎实有效。认真落实九个防范化解重大风险工作方案，全力以赴打好风险防控攻坚战。债务风险化解成效明显。制定完善债务风险化解规划和应急处置预案，对政府债务风险预警地区加强管控，坚决遏制不规范举债行为。强化“因债施策”有序化解存量债务，全年通过债券置换、借新还旧、预算偿债等方式化解债务123.5亿元，全市政府债务风险总体可控，高坪、南部先后退出财政重整。多措并举综合施策，严控政府隐性债务风险。金融风险防范力度加大。全面开展全市金融风险大排查，强力推进互联网金融风险专项整治，严厉打击非法集资活动，守住了不发生区域性、系统性金融风险的底线，全市金融安全区建设综合考评列全省第4位。安全稳定风险管控有力。扎实抓好安全生产，全年未发生较大及以上生产安全事故。食品药品安全形势稳中向好，非洲猪瘟疫情防控扎实有效。全力做好信访稳定工作，深入开展扫黑除恶专项斗争，一批积案老案得到依法妥善解决。全市信访总人次下降15.6%，刑事案件数下降13.8%，群众对平安建设的满意度不断提升。扎实抓好防汛减灾各项工作，成功应对近年来嘉陵江干流最大洪峰过境。

　　（七）着力转职能优服务，自身建设得到加强。 严明政治纪律和政治规矩，牢固树立“四个意识”，切实增强“四个自信”，坚决做到“两个维护”。常态化制度化推进“两学一做”学习教育，扎实开展“大学习、大讨论、大调研”活动，法治、诚信、开放、效能、廉洁政府建设取得新成效。办理市人大代表建议297件、政协委员提案335件，办结率100%；法治政府建设在全国100个被测评城市中排第25位、较上一年上升8位，蝉联西部17个地级市首位，“社会公众满意度调查”得分连续两年居被测评城市第一。强化政府诚信引领，推进社会信用体系建设，公示涉企信用信息总量居全省第2位，城市综合信用指数稳居全省“一方阵”。深化“放管服”改革，市本级行政许可事项减至280项，93%的行政许可事项、91%的公共服务事项实现“最多跑一次”，一体化政务服务平台按时办结率达100%。坚决整治形式主义、官僚主义“新表现”，严防“四风”问题反弹回潮；扎实推进政府系统党风廉政建设，全面加强审计监督，坚决防范各类腐败行为，风清气正的良好政治生态得到巩固发展。

　　与此同时，第四次全国经济普查全面启动，国防动员、人民防空、双拥优抚、军民融合等工作取得新成效，国资监管、统计调查、检验检疫、机关事务、国土、质监、海关、气象、档案、地方志、保密、国安、邮政、贸促、驻市外机构、外事侨务、港澳台等工作取得新进展，民族宗教、精神文明、慈善、老龄、妇女儿童、青少年、残疾人、工会、红十字、爱国卫生等事业有了新进步。

　　各位代表！最是奋斗动人心。过去一年，我们干成了许多大事、办好了许多实事，推动南充发生了令人振奋的新变化，经济实力更强了，发展质量更高了，生态环境更好了，城乡面貌更美了，群众获得感幸福感安全感更实了。这些成绩，是省委、省政府和市委坚强领导的结果，是市人大、市政协和人大代表、政协委员监督支持的结果，是全市广大干部群众共同努力、苦干实干的结果。在此，我代表市人民政府，向全市人民，向各位代表、各位委员，向各民主党派、工商联、无党派人士和各人民团体，向驻市解放军、武警官兵和政法干警，向所有关心支持南充建设发展的各界人士和海内外朋友们表示衷心的感谢和崇高的敬意！

　　在肯定成绩的同时，我们也清醒认识到，全市经济社会发展中还存在一些问题和不足：高质量发展有待进一步推进，产业实力不强、内在不优的情况仍比较明显；改善民生力度有待进一步加大，精准脱贫、防止返贫和全面小康的任务依然艰巨繁重，公共服务水平还需提高；政府效能有待进一步加强，营商环境还需更加优化，等等。对此，我们一定高度重视，直面问题，采取有力措施，认真加以解决。

二、2019年工作安排

　　各位代表！追梦新时代，奋进新征程，展示新作为。市委六届八次全会全面贯彻落实省委“一干多支、五区协同”等战略部署，坚持一张蓝图绘到底、与时俱进谱新篇，进一步丰富完善了“155发展战略”内涵，鲜明发出建设成渝第二城、争创全省经济副中心的时代号令，这是继往开来、接续奋斗的科学抉择，也是乘势而上、勇开新局的责任担当，为推动治南兴南再上新台阶指明了前进方向和路径遵循。我们一定要保持定力、坚定信心，咬定目标不放松、聚精会神抓落实，奋力实现大城崛起的美好蓝图！市委六届九次全会明确了2019年全市工作总体思路、着力重点和具体要求，进一步为我们列出了任务书、挂出了作战图、发出了动员令。全市上下要对标对表、细化实化，撸起袖子加油干、一马当先抓推进，加快构建“5+5”现代产业体系，大力实施“5+100”重大工程项目，推动形成“一主一副、区域协同”发展新格局，共同谱写南充发展的崭新篇章！

　　今年政府工作的总体要求是：坚持以习近平新时代中国特色社会主义思想为指导，全面贯彻党的十九大和十九届二中、三中全会、中央经济工作会议精神，深入学习贯彻习近平总书记对四川工作系列重要指示精神，认真落实省委十一届三次、四次全会、省委经济工作会议和市委六届八次、九次全会、市委经济工作会议决策部署，统筹推进“五位一体”总体布局，协调推进“四个全面”战略布局，坚持稳中求进工作总基调，坚持新发展理念，坚持推动高质量发展，坚持以供给侧结构性改革为主线，坚持深化市场化改革、扩大高水平开放，聚焦总量“三突破”、增速“三高于”、排位“一方阵”总目标，按照市委坚定不移打好“三场攻坚战”“三场突围战”“三场持久战”和“产业年、项目年、开放年”总部署，深入实施“155发展战略”，统筹推进稳增长、促改革、调结构、惠民生、防风险、保稳定工作，进一步稳就业、稳金融、稳外贸、稳外资、稳投资、稳预期，保持经济持续健康发展和社会大局稳定，为全面建成小康社会收官打下决定性基础，以优异成绩庆祝中华人民共和国成立70周年。

　　全年经济社会发展主要预期目标是：地区生产总值增长8%以上，全社会固定资产投资增长12%以上，一般公共预算收入增长8%以上，规上工业增加值增长10.5%以上，社会消费品零售总额增长11.8%以上，服务业增加值增长9%以上，城镇和农村居民人均可支配收入分别增长8.6%、9.5%以上，城镇登记失业率控制在4.4%以内。

　　围绕上述目标，今年重点抓好九个方面的工作。

　　（一）牢固树立底线思维，坚决打好防范化解重大风险攻坚战

　　防范化解金融风险。进一步健全监测预警和早期干预机制，坚决遏制金融市场风险累积。加大民间借贷风险监控力度，加强投资人教育，严厉打击校园贷、套路贷、非法集资、乱办金融等违法违规金融活动。加强对互联网金融等新兴金融业态的监管，深入推进网贷机构清理，平稳有序做好处置工作。切实强化信用风险管控，依法打击恶意逃废金融债务行为，支持银行机构加快处置不良资产。深入推进南充金融安全区建设，不断优化金融生态环境。

　　防范化解债务风险。 坚持举债有度、限额管理、严防风险“三条原则”，加强对市、县政府债务和隐性债务的统计监测，坚决遏制不规范举债、变相举债等行为。加大存量债务偿还力度，争取借新还旧债券资金60亿元以上，确保政府债务余额控制在省政府核定的限额内，力争9县（市、区）全部退出风险预警或风险提示名单。严密防控企业债务风险，支持企业通过盘活闲置资产等方式优化资产债务结构。

　　防范化解房地产市场风险。 坚持“房住不炒”原则，进一步强化市场监测和调控，促进房地产市场平稳健康发展。加大“商改住”去库存力度，鼓励固定职业农民工、新就业大学生等新市民进城购房，确保商品房待售面积、库存消化周期“双下降”。深入开展市辖城区住房租赁试点，积极培育发展住房租赁市场特别是长期租赁，满足不同群体住房需求。严格执行差别化住房信贷政策，满足首套刚需、支持改善需求、遏制投机炒房。持续抓好市场监管整顿，严厉查处开发、销售、中介违规行为。优化土地资源配置，强化供地后续监管。切实加强“风险楼盘”整治，妥善处置一批房地产开发遗留问题。

　　防范化解安全和稳定风险。严格落实安全生产责任制，深入开展“九大专项整治行动”，坚决遏制重特大事故发生。大力实施食品药品安全放心工程，积极争创国家食品安全示范城市，突出抓好校园食品安全专项整治。持续加强非洲猪瘟疫情防控。加强社会治安防控体系建设，纵深推进扫黑除恶专项斗争，扎实抓好反恐防暴、反邪教、禁毒斗争等重点工作，努力建设更高水平的“平安南充”。强化社会矛盾排查调处，加大信访积案化解处理力度。建立高效科学的灾害防范体系，加强应急演练和救援力量建设，提高应急处置实战化水平。

　　（二）全面落实精准方略，坚决打好精准脱贫攻坚战

　　提高脱贫质量。聚焦“两不愁、三保障”，坚决完成73个贫困村退出、1.32万贫困人口脱贫和全市巩固提升“2+1”年度任务。加大产业扶贫和就业扶贫力度，推动脱贫奔康产业园提质增效，确保贫困家庭劳动力新增转移就业7000人以上。统筹好贫困村与非贫困村发展、贫困群众与临界贫困群众帮扶。深入开展“回头看”“回头帮”，强化对摘帽县和脱贫户后续扶持。持续抓好扶贫领域作风建设，坚决防止数字脱贫、虚假脱贫、形式脱贫。

　　补齐基础短板。深入实施好22个扶贫专项年度方案，完成易地扶贫搬迁318人、农村危房改造3.5万户、地灾避险安置340户，巩固提升26.1万农村居民饮水安全。加强路水电气宽带“五网”建设，进一步改善群众生产生活条件。加大贫困学生入学和控辍保学力度，确保适龄儿童义务教育不辍一人。深入开展健康扶贫“五大行动”，全面落实“十免四补助”。用好用活“四项基金”，确保发挥应有作用。

　　抓实扶贫协作。 扎实抓好与金华、温州、台州3市扶贫协作和省内对口帮扶工作，全面落实已签合作协议，用好对口帮扶资金，建好扶贫协作项目。深化拓展合作领域，推动更多南充产品进入东部市场，大力引进优质企业来南充投资兴业。加强跨区域人力资源合作，积极引进一批创业创新人才、输出一批劳务人员。推动教育、医疗等领域人才交流，扩大社会机构、志愿服务组织等结对帮扶范围。

　　（三）夯实绿色生态本底，坚决打好污染防治攻坚战

　　强力整治环境问题。持续打好污染防治“八大战役”，确保主城区PM2.5浓度较基准年下降17%以上，国、省考核地表水质断面优良比例达到100%。扎实做好第三次国土调查、第二次全国污染源普查，全面完成“大棚房”和重点区域违规违法占用国家资源挤占生态空间问题专项清理整治。突出抓好中、省环保督察及“回头看”反馈问题整改，坚决确保按期销号。

　　全力加强生态建设。严格落实生态红线刚性保护，扎实推进嘉陵江流域生态文明先行示范区、嘉陵江（南充段）绿色生态走廊建设。充分发挥河（湖）长制应有作用，大力实施“清河、护岸、净水、保水”四项行动，全面完成主要河流河道管理范围划定，突出抓好嘉陵江综合保护和小流域治理。加强天然林保护，完成新一轮退耕还林还草3万亩以上。深入实施大规模绿化南充行动，确保全市森林覆盖率提升至40.78%。

　　推动绿色低碳发展。严格实行能源和水资源消耗、建设用地等总量和强度“双控”，提高资源节约和循环利用水平。加快建设国家级资源循环利用基地，推动南充经开区、阆中经开区、蓬安工业园区实施园区循环化改造。大力发展清洁生产，鼓励企业实施锅炉、电机等重点用能装备节能改造，引导企业降低能耗、物耗和污染物排放水平。积极推广应用新能源汽车，倡导简约适度、绿色低碳生活方式，深入开展绿色机关、绿色校园、绿色社区创建。确保全市单位GDP能耗下降2%以上。

　　（四）大力实施“产业年”行动，持续壮大现代产业集群

　　持续壮大汽车汽配产业。开工建设律奥汽配、睿辉汽配等项目，加快推进吉利二期、重庆银翔等项目，确保博萨汽配、宁靓重钢等项目竣工投产，推动吉利一期、中国重汽等项目扩大产能。启动建设汽车文化小镇。大力推进延链补链、配套发展，引进汽车汽配项目10个以上。确保整车产量突破1.5万台，汽车汽配产业产值增长14.1%以上、突破800亿元。

　　持续壮大油气化工产业。开工建设禾本科技、凯伦新材料等项目，加快鑫达高分子功能母料等项目建设进度，推动青龙化工、顺城盐化等项目竣工投产，确保四川能投PTA项目开车达产、尽快发挥龙头引领作用。力促石达化工、联成化学等项目扩能增产。引进重大油气化工项目4个以上。确保油气化工产业产值增长23%以上、突破400亿元。

　　持续壮大丝纺服装产业。 加快推进富安娜二期、新安佳新型再生纤维等项目，确保银河地毯搬迁、金泰纺织等项目全面竣工，推动六合集团、嘉美印染等重点企业加大技改力度，进一步提升市场竞争能力。精心筹办2019中国西部丝绸博览会，持续扩大“中国绸都”影响力。新（改）建蚕桑基地4万亩以上。确保丝纺服装产业产值增长15%以上、突破500亿元。

　　持续壮大现代物流产业。 启动建设中通快递、丰树物流等项目，加快推进新成储川东北物流中心、臻信粮油物流等项目，确保南充港冷链物流中心、花都商贸物流中心等项目建成运营。引进知名物流企业18家以上，新增A级物流企业3家以上。加快6个县级物流节点建设，新增城乡配送网点300个以上。确保现代物流产业产值增长16.8%以上、突破530亿元。

　　持续壮大现代农业产业。深化农业供给侧结构性改革，有序发展晚熟柑桔、优质粮油等特色主导产业，建成县级以上现代农业园区21个以上、特色产业基地25万亩以上。加快推进农业“接二连三”融合发展，大力发展农产品精深加工，实现农产品电商销售额突破10亿元。做强“好充食”等农产品区域品牌，新增“三品一标”农产品20个以上。落实粮食安全省长责任制部署要求，抓实抓好粮食安全保障。扎实推进亭子口灌区前期工作，加快建设升钟灌区二期等水利工程，建成高标准农田32万亩以上。确保现代农业产业产值增长9.3%以上、突破1100亿元。

　　持续壮大 “五大百亿战略性新兴产业”。加快发展电子信息产业，启动建设锦创宏光电、图灵通诺结算机器人等项目，加快建设中科九微、华讯方舟等项目，确保三环六期、宽带乡村等项目投产投用；实施大数据应用示范工程，推动川东北区域云计算中心建设，形成数字经济新增量；力争电子信息产业产值增长13%以上、突破70亿元。加快发展新材料产业，大力推进中远二期、中城建新型建筑材料等项目，确保蜀爱新材料、家旺钢化等项目竣工投产，培育发展新材料研发、设计、检测、认证和服务推广等新业态；力争新材料产业产值增长25%以上、突破70亿元。加快发展高端装备制造产业，扎实推进明辉机电、零零昊真空技术应用等项目，推动九天真空实施智能化改造，充分发挥西充军民融合高端装备制造产业园的集聚引领作用，大力引进航空航天、民用机械、轨道交通等高端装备及配套产业；力争高端装备制造产业产值增长13%以上、突破35亿元。加快发展生物医药产业，启动建设四川弘达化学原料药基地、新绿色中药材种植加工基地，推动四川景弘、香雪制药等企业扩能增产；依托川北医学院等科研院校优势，支持企业深化产学研合作，推动生物技术药、生物制剂取得新突破；力争生物医药产业产值增长21%以上、突破90亿元。加快发展节能环保产业，加快实施节能环保重点工程，扎实抓好顺高嘉、阆南西、仪营蓬三大片区固废处理循环产业园建设，大力推进国能生物、嘉源环保等项目；努力在做优高效节能产业、做大先进环保产业、做精资源循环利用产业、做强节能环保服务业上取得更大成效；力争节能环保产业产值增长13%以上、突破35亿元。

　　持续壮大文化旅游产业。深入挖掘南充丰富文化内涵，充分发挥丝绸文化、红色文化、三国文化、春节文化等独特资源优势，创新开发一批特色鲜明的文化主题旅游项目。加快建设纵横文旅、宜华康养城、阆中赛城等“十大文旅项目”，谋划实施一批文化街区、文化园区等重点工程。创新体制机制，支持文艺院团发展，引导扶持文艺精品创作。大力实施“文化+”“旅游+”工程，发展壮大时尚设计、工业设计、建筑设计等新业态。扎实抓好旅游市场营销，深度融入“成渝西”黄金旅游带，打造一批精品文化旅游线路。以阆中古城和朱德故里两个5A级景区为龙头，带动全市旅游景区品质提升，全年新创建3A级以上旅游景区3个。全市旅游总收入增长16.1%以上、突破650亿元，文化旅游产业产值增长15%以上、突破900亿元。

　　（五）大力实施“项目年”行动，扎实推进重大工程项目

　　扎实推进城市建设项目。实施重大项目189个，完成年度投资558亿元以上。开工建设将军路嘉陵江大桥、金融大道延长线等项目，启动实施阳春路、都尉路等城市道路综合改造，推动丝绸小镇、清泉寺文化小镇等特色街区建设，加快建设“双子塔”、清晖阁等地标项目，扎实推进高铁商务区、燕儿窝中央商务区、城市中央森林公园等项目，确保北湖片区改造提升工程全面竣工。大力实施“六大工程”，开工公共服务配套项目100个以上，建设地下综合管廊19公里以上。稳步推进“城市双修”，启动编制全市国土空间规划。

　　扎实推进交通枢纽项目。实施重大项目82个，完成年度投资104亿元以上。力争成南达万高铁、汉巴南铁路年内开工。开工建设成南扩容和阆仪营、南潼、南充过境高速公路，加快建设营达高速公路和顺蓬营等干线公路。按照国家开放口岸机场要求，高标准建设高坪机场新航站楼；开工建设阆中机场。启动实施南充海事基地等项目，协同推进利泽航电枢纽建设。

　　扎实推进教科文卫项目。实施重大项目55个，完成年度投资176亿元以上。开工建设顺庆大学城、南充高中北部新城校区和高坪校区等项目，加快推进嘉陵江文化旅游职业学院等项目。开工建设三环研究院，加快实施南充生物医药研究院、蚕桑丝绸研究中心提升工程。大力推进嘉陵江文化综合保护开发项目，确保川东北文化传媒中心二期竣工投用。启动筹建市中医医院，加快建设市中心医院江东院区、川北医学院国际医院等项目。

　　扎实推进旅游三产项目。实施重大项目103个，完成年度投资615亿元以上。深入实施旅游“双百亿工程”，加快推进南部升钟湖旅游度假区提升、蓬安相如故城保护开发等项目建设，确保西河生态乡村旅游等项目基本建成。深入实施服务业“三百工程”，开工建设南充国际会展中心、西巷文化商业街等项目，大力推进国际购物小镇、奥特莱斯广场等项目，确保当当网农村电商全国总部等项目建成投用。加快建设川东北金融中心，鼓励金融机构总部集聚发展，引进金融及相关机构20家以上。

　　扎实推进产业园区项目。实施重大项目36个，完成年度投资92亿元以上。加快建设南充新能源汽车产业园、航空港电子信息产业园等特色园区，推动南充高新技术产业园争创国家级高新区。加快建设川东北有机农产品精深加工园，大力提升国家现代农业示范区业态层次，确保中法农业科技园全面开园、完美亮相，推动国家农业科技园区争创国家级示范区。继续加大南充现代物流园产业填充力度，做强国家级示范物流园区实力。

　　扎实推进 “100件大事”。 始终把“100件大事”作为贯穿和引领全市当前及今后一个时期经济社会发展的重心所在、关键所在、着力所在，认真落实一事一主体、一主体一责任人要求，强化目标导向，把握关键节点，严卡工程进度，推动市本级25个支撑项目完成投资62亿元以上，9县（市、区）186个支撑项目完成投资1034亿元以上。强化问题导向，通过财政资金优先安排、专项资金优先整合、信贷资金优先投放、社会融资优先推介，多渠道破解资金瓶颈；实行市级统筹指标重点支持、新增建设用地指标重点倾斜，切实加大调剂力度、保障“100件大事”用地需求。强化效果导向，坚持立说立行、善作善成，坚决按照市委决策部署，围绕“100件大事”的年度目标，提振“精气神”、干好“精细活”，推动在建项目加快进度、建成项目全新展示，让群众实实在在感受到南充发展的新成效、新变化。

　　（六）大力实施“开放年”行动，全面提升开放合作水平

　　不断深化区域合作。切实加强与长三角、珠三角、京津冀、粤港澳大湾区、北部湾经济区等重点区域的对接合作。主动承接成渝两地产业转移和功能疏解，落实成南“1+N”区域协同发展合作协议，抓好深度融入重庆发展。加快川东北区域协同发展步伐，强化与达州“双核”联动、与广安融渝协同、与广元生态共建、与巴中发展互惠，力促川东北9个市际合作协议落地落实。协同推进阆苍南一体化发展。

　　稳定外资外贸增长。加快建设南充跨境电商平台、进出口综合服务平台，着力提升南充公用型保税仓运营水平。积极申建保税物流中心（B型）、四川自贸试验区协同改革先行区、跨境电商示范区。大力推动多式联运和通关便利化，积极推广国际贸易“单一窗口”应用，提升外贸进出口监管效率，降低企业运营成本。深度挖掘“四向拓展、全域开放”机遇，积极拓展南亚、东南亚等新兴国际市场，支持优势产业、优秀企业、优质产品走出去。全面落实准入前国民待遇加负面清单管理制度，健全外商来南投资的服务保障体系，营造内外资企业一视同仁、公平竞争的公正市场环境。确保全年实现进出口总额10亿元以上、增长10%以上，实际利用外资3.5亿元以上。

　　继续强力招大引强。 深度对接国、省产业政策、投资导向和支持重点，紧盯“三类500强”和行业领军企业，精准推介、定向攻关，全力引进一批大企业、大项目、大集团。积极参加中外知名企业四川行、西部投资大会等全省重大投资促进活动，精心举办北京、福建、山东等地专题招商及会展活动，确保取得更多实质性成果。拓展延伸招商触角，支持筹建厦门、沈阳和南非（安哥拉）南充商会。全年招引5亿元以上产业项目超过100个，到位资金700亿元以上。

　　着力壮大民营经济。落实全省“营商环境提升年”部署，打造更加公平透明的营商环境。发挥民营经济产业发展基金引导作用，支持企业产品创新、技术创新、商业模式创新。构建民营企业融资服务平台，引导金融机构加大信贷投放，扩大中小企业“应急转贷资金”使用范围和规模，着力缓解民营企业融资难融资贵问题。进一步构建亲清新型政商关系，畅通企业反映诉求渠道，依法保护其合法权益；建立领导干部联系重点企业、商（协）会制度，健全产需对接、科技成果对接等服务体系，千方百计帮助民营企业解忧纾困。鼓励和引导民营资本参与基础设施领域“补短板”。全年新增市场主体3万户以上，民营经济增加值增长8.1%以上、突破1300亿元。

　　（七）持续深化改革创新，加速集聚激发内生动能

　　深化供给侧结构性改革。认真贯彻“巩固、增强、提升、畅通”八字方针，坚持去产能与优产能相结合，依法处置一批“僵尸企业”，推动过剩产能加快出清；支持重点企业技术改造，完成技改投资增长10%以上；积极培育人工智能、信息终端、云计算、物联网等新业态，推动数字经济与实体经济融合发展。落实更大规模减税、更明显降费，切实减轻企业负担。扎实开展质量提升行动，新增注册商标1200件以上。

　　深入实施重点领域改革。加快国资国企改革，做大做强做优国有资本，依法科学管理国资国企。大力推进地方金融改革，以服务实体经济为导向，支持发展民营银行和社区银行。深入推进公立医院改革，确保个人卫生支出占卫生总费用的比例降到30%以下。加快推进城乡“五个一体化”和改革助推乡村振兴两大示范项目，扎实抓好国家新型城镇化试点等国、省改革试点，统筹推进财税制度、农业农村和砂石资源经营管理体制等重点改革，完成市、县政府机构改革。

　　大力推进创新驱动发展。加快建设国家创新驱动发展助力工程示范市、国家知识产权试点示范市，积极争创省级创新型城市。扎实推进南充双创中心等平台建设，推动航空港科技创新中心全面完工。深入实施“嘉陵江英才工程”和“三奔计划”，引进硕士研究生以上学历、稀缺性专业、高级职称等高层次创新创业人才1000名以上、团队10个以上，申请国家发明专利500件以上。强化科技协同创新，加快推进川东北军民融合产业园建设。力争科技对经济增长贡献率达到52%以上。

　　（八）坚持城乡区域协调，培育壮大县域经济实力

　　支持县域经济发展。按照“顺高嘉同城发展、南西蓬一体发展、阆南仪协作发展、营山融渝发展”总布局，大力支持县域优势产业发展和重大项目建设，加快推动县域经济特色发展、错位发展。重点支持顺庆、南部等地做大做强经济实力，积极争创“县域经济强县”；支持阆中、仪陇等地培育壮大文化旅游产业，积极争创“天府旅游名县”；支持蓬安、西充等地加快绿色发展，积极争创“生态建设标杆县”；支持高坪、嘉陵、营山等地加速壮大实体经济，积极争创“县域经济先进县、进步县”。

　　加快城镇建设步伐。不断完善市域城镇体系，优化城镇布局，有序推进县城建设和功能配套，加强小城镇基础设施建设和人居环境整治，着力提高精细化管理水平。深入推进“百镇建设行动”试点镇建设，确保创建国、省级特色小镇各1个。深化扩权强镇改革，推动综合执法等管理权限有序下沉。稳步推进农业转移人口市民化，着力解决转户居民城镇养老、医疗、教育等问题，力争全市常住人口城镇化率提高1.4个百分点。

　　扎实抓好乡村振兴。聚焦“五个振兴”目标，落实好我市乡村振兴三年行动方案，建成市级以上乡村振兴先进乡镇10个、示范村50个。大力发展多种形式适度规模经营，引进年营业额超2000万的农业龙头企业10家以上，培育市级以上农民合作示范社20家以上、新型职业农民2000人以上。扎实推进农村人居环境整治，大力实施村容村貌“六化”工程，新（改）建农村公路1000公里以上、乡镇污水处理厂80座以上，改造农村户厕5.1万户以上。加强农村公共文化建设，积极开展“新家园、新生活、新风尚”活动，新建村级综合文化服务中心70个以上。鼓励和引导城市工商资本有序参与乡村建设，支持各类人才返乡创业，促进资源要素向乡村流动、发挥积极作用。

　　（九）突出抓好民生改善，努力增进人民群众福祉

　　大力办好民生实事。坚持民生支出只增不减，扎实做好普惠性、基础性、兜底性工作，全面完成30件民生实事。落实“菜篮子”市长负责制。加快建设市第四水厂等民生项目，确保荆溪污水处理厂一期、嘉东污水处理厂二期建成投用。完成城镇危旧房棚户区改造3.9万套。新增城镇就业6万人以上。落实退役军人待遇保障，完善退役士兵基本养老、基本医疗保险接续政策。加大农民工服务保障和农村“三留守”人员关爱力度。进一步完善社会救助体系。大力实施全民参保计划，推进社会保险人员全覆盖。

　　全面发展社会事业。优先发展教育事业。支持西华师范大学创建博士学位授予单位、西南石油大学（南充）创建双一流大学和百年名校、川北医学院创办高水平医科大学；提高义务教育均衡化水平，推进高中阶段教育普及攻坚，办好学前教育，提升职业教育、民办教育、特殊教育、继续教育质量，着力解决中小学课外负担重等问题；强化国防教育。切实加大科普力度，提高公众科学素养。广泛开展文化惠民活动，精心筹办第四届嘉陵江灯戏暨地方戏剧艺术节。深入推进全民健身运动。加快建设“健康南充”，新增医疗床位1500张以上，适老化改造养老床位2200张以上，努力满足群众多层次多样化医疗健康需求。

　　切实加强社会治理。纵深推进“法治南充”建设，扎实抓好“七五普法”，深入开展“法律七进”，大力推进公共法律服务体系建设，持续深化基层法治示范创建。加强思想道德建设和群众性精神文明创建，提高全社会文明素质。健全社会心理服务体系，培育自尊自信、理性平和、积极向上的社会心态。学习借鉴新时代“枫桥经验”，加强源头治理和网格化管理，推动社会治理重心下移。

三、大力实施“政府效能提升年”行动

　　各位代表！责重如山看担当，打铁还须自身硬。新的一年，我们将坚持把政治建设摆在首位，深入推进“两学一做”学习教育常态化制度化，扎实开展“不忘初心、牢记使命”主题教育，进一步树牢“四个意识”、坚定“四个自信”，坚决做到“两个维护”，自觉在思想上政治上行动上同以习近平同志为核心的党中央保持高度一致，坚决确保党中央国务院、省委省政府和市委决策部署不折不扣落到实处。强化法治思维，坚持依法行政，自觉接受市人大及其常委会的法律监督、工作监督和市政协的民主监督，主动接受监察监督、社会监督和舆论监督，持续加强审计监督，支持开展司法监督。健全“政府承诺+社会监督+失信问责”机制，以政府诚信示范引领全社会诚信建设。全面落实中央八项规定、省委省政府十项规定及其实施细则精神，认真执行市委市政府九项规定及其实施细则，驰而不息纠正“四风”。严格履行全面从严治党主体责任和“一岗双责”，坚决落实省委“六个深刻汲取、六个问问”和市委“三问三查三想”部署要求，保持警钟长鸣，确保见行见效；深入推进政府系统党风廉政建设，持续巩固风清气正的良好政治生态。

　　各位代表！行政效能是政府工作的生命线。新的一年，我们将大力实施“政府效能提升年”行动，以深化“放管服”改革为牵引，推动政府职能加快转变，行政效能明显提高，公信力和执行力不断增强，努力建设人民满意政府，更好满足群众对美好生活的新期待。

　　（一）聚焦“简政放权”，全面提升审批效能。 进一步完善政府部门权责清单，清理规范各类行政权力、公共服务、涉审中介服务等事项。大力实施工程建设项目审批制度改革，继续优化企业登记、施工许可审批等流程，全面推行“联合评审”“联合踏勘”“联合图审”“联合验收”，确保全流程审批用时压缩至120个工作日以内。深化商事制度改革，统筹实施“证照分离”“多证合一”改革，确保企业开办时间压缩至3个工作日以内。

　　（二）聚焦“便利快捷”，全面提升服务效能。加快推进“最多跑一次”改革，梳理公布第二批“最多跑一次”事项清单，力争全市政务服务事项全部实现“最多跑一次”。深化“三集中、三到位”改革，实现办事只进一扇门、只去一个窗。大力推行“互联网+政务服务”，加快推进“一网通办”，拓展全流程网上办理事项范围，打造“24小时不打烊”的在线服务大厅，确保全市80%以上政务服务事项网上可办。持续推进“减证便民”，大力推行代办服务，更好满足企业和群众需求。加大政务公开力度。建立政务服务“好差评”制度，让企业和群众评判服务绩效。

　　（三）聚焦“循环顺畅”，全面提升运行效能。 扎实抓好政府机构改革后续工作，促进部门职能衔接、人员融合，畅通循环通道，进一步优化协同高效的机构职能体系。健全市、县政府之间协作配合机制，切实增强工作协同性、连贯性；重新划分市、区两级财权事权，进一步厘清市、区权责关系。大力抓好精文减会，确保全市政府系统文件、会议均减少30%以上。着力提升电子政务水平，推广应用OA办公系统，进一步缩短公文运转周期。切实解决报表材料过多过滥问题，让干部腾出更多时间和精力推动工作。

　　（四）聚焦“高效有力”，全面提升执行效能。大力推行清单化、项目化管理，紧扣上级决策部署，建立重点任务工作台账，做到一个专项、一套方案、一抓到底。严格执行决策部署和交办事项动态跟踪、回销报告制度，切实做到言必行、行必果、果必复。认真落实“六个一”调度机制、“三分法”推进机制、“六联动”考评机制，全力推动各项工作“得满分、争第一、一方阵”。全面落实市委“133”系列政策措施，充分激发干部担当作为。创新政务督查方式，严控总量和频次，提高督查实效性。坚决治理“庸懒散浮拖”，打通执行落实“最后一公里”。

　　（五）聚焦“公平公正”，全面提升监管效能。健全完善事中事后监管体系，推进“双随机、一公开”跨部门联合监管、“互联网+监管”，做到“一次检查、全面体检”；探索新兴产业包容审慎监管，给新业态新模式成长留出空间。深化综合行政执法改革，推行行政执法“三项制度”，促进严格规范公正文明执法；优化安全生产、生态环保等领域执法方式，对违法者依法严惩、对守法者无事不扰。

　　各位代表！积力所举，无往不胜；众智所为，无所不成。让我们更加紧密地团结在以习近平同志为核心的党中央周围，高举中国特色社会主义伟大旗帜，以习近平新时代中国特色社会主义思想为指导，在省委、省政府和市委的坚强领导下，不忘初心、牢记使命，锐意进取、真抓实干，加快建设成渝第二城，全力争创全省经济副中心，以优异成绩向新中国70华诞献礼！
